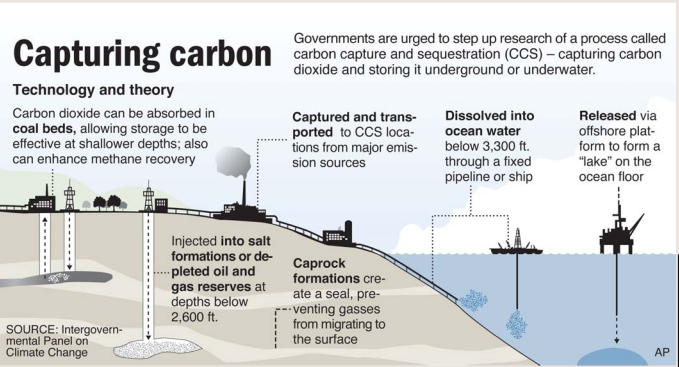# Carbon Capture & Storage: Blue-Sky Technology or Just Blowing Smoke?

**DOI:** 10.1289/ehp.115-a538

**Published:** 2007-11

**Authors:** Charles W. Schmidt

Towering 650 feet over the sea surface and spouting an impressive burning flare, it would be easy to mistake the Sleipner West gas platform for an environmental nightmare. Its eight-story upper deck houses 200 workers and supports drilling equipment weighing 40,000 tons. Located off the Norwegian coast, it ranks among Europe’s largest natural gas producers, delivering more than 12 billion cubic feet of the fuel annually to onshore terminals by pipeline. Roughly 9% of the natural gas extracted here is carbon dioxide (CO_2_), the main culprit behind global warming. But far from a nightmare, Sleipner West is actually a bellwether for environmental innovation. Since 1996, the plant’s operators have stripped CO_2_ out of the gas on-site and buried it 3,000 feet below the sea floor, where they anticipate it will remain for at least 10,000 years.

We believe [CCS] is a viable way to cut global warming pollution. . . . We have the knowledge we need to start moving forward.

–David Hawkins, Natural Resources Defense Council

Operated by StatoilHydro, Norway’s largest company, Sleipner is among the few commercial-scale facilities in the world today that capture and bury CO_2_ underground. Many experts believe this practice, dubbed carbon capture and storage (sometimes known as carbon capture and sequestration, but in either case abbreviated CCS), could be crucial for keeping industrial CO_2_ emissions out of the atmosphere. Sleipner injects 1 million tons of CO_2_ annually into the Utsira Formation, a saline aquifer big enough to store 600 years’ worth of emissions from all European power plants, company representatives say.

With mounting evidence of climate change—and predictions that fossil fuels could supply 80% of global energy needs indefinitely—the spotlight on CCS is shining as brightly as the Sleipner flare. A panel of experts from the Massachusetts Institute of Technology (MIT) recently concluded that CCS is “the critical enabling technology to reduce CO_2_ emissions significantly while allowing fossil fuels to meet growing energy needs.” The panel’s views were presented in *The Future of Coal*, a report issued by MIT on 14 March 2007.

Environmental groups are split on the issue. Speaking for the Natural Resources Defense Council (NRDC), David Hawkins, director of the council’s Climate Center and a member of the MIT panel’s external advisory committee, says, “We believe [CCS] is a viable way to cut global warming pollution. . . . We have the knowledge we need to start moving forward.” Other environmental groups, including the World Resources Institute, Environmental Defense, and the Pew Center on Global Climate Change, have also come out in support of CCS. These groups view CCS as one among many alternatives (including renewable energy) for reducing CO_2_ emissions.

Greenpeace is perhaps the most vocal critic of CCS. Truls Gulowsen, Greenpeace’s Nordic climate campaigner, stresses that CCS deflects attention from renewable energy and efficiency improvements, which, he says, offer the best solutions to the problem of global warming. “Companies are doing a lot of talking about CCS, but they’re doing little to actually put it into place,” he says. “So, they’re talking about a possible solution that they don’t really want to implement now, and at the same time, they’re trying to push for more coal, oil, and gas development instead of renewables, which we already know can deliver climate benefits.”

## Coal Use Drives CCS Adoption

The pressure to advance on CCS has been fueled by soaring coal use worldwide. China, which is building coal-fired power plants at the rate of two per week, surpassed the United States as the world’s largest producer of greenhouse gases in June 2007, years earlier than predicted. Coal use in India and other developing nations is also on the rise, while the United States sits on the largest coal reserves in the world, enough to supply domestic energy needs for 300 years, states the MIT report. Coal already supplies more than 50% of U.S. electricity demand and could supply 70% by 2025, according to the International Energy Agency. Meanwhile, coal-fired power plants already account for nearly 40% of CO_2_ emissions worldwide, a figure that—barring some dramatic advance in renewable energy technology—seems poised to rise dramatically. During a 6 September 2007 hearing of the House Select Committee on Energy Independence and Global Warming, Chairman Edward Markey (D–MA) noted that more than 150 new coal plants are being planned in the United States alone, with another 3,000 likely to be built worldwide by 2030.

A mature CCS system would capture, transport, and inject those emissions underground to depths of at least 1 km, where porous rock formations in geologically favorable locations absorb CO_2_ like a sponge. At those depths, high pressures and temperatures compress the gas into a dense, liquid-like “supercritical” state that displaces brine and fills the tiny pores between rock grains. Three types of geological formations appear especially promising for sequestration: saline (and therefore nonpotable) aquifers located beneath freshwater deposits; coal seams that are too deep or thin to be extracted economically; and oil and gas fields, where CO_2_ stripped from fuels on-site can be injected back underground to force dwindling reserves to the surface, a process called “enhanced recovery.” Using CO_2_ for enhanced recovery has a long history, particularly in southwestern Texas, where oil yields have been declining for decades.

Of these three options, saline aquifers—with their large storage capacity and broad global distribution—are considered the most attractive. Thomas Sarkus, director of the Applied Science and Energy Technology Division of the DOE National Energy Technology Laboratory (NETL), suggests saline aquifers in the central United States could conceivably store 2,000 years’ worth of domestic CO_2_ emissions.

Apart from Sleipner, only two other industrial-scale CCS projects are in operation today. In Algeria, a joint venture involving three energy companies—Statoil-Hydro, BP, and Sonatrach—stores more than 1 million tons of CO_2_ annually under a natural gas platform near In Salah, an oasis town in the desert. And in Weyburn, Canada, comparable volumes are being used by EnCana Corporation, a Canadian energy company, for enhanced recovery at an aging oil field. The CO_2_ sequestered at Weyburn comes by pipeline from a coal gasification plant in Beulah, North Dakota, 200 miles away. Unlike other enhanced recovery projects—wherein the ultimate fate of CO_2_ is not the primary concern—Weyburn combines fossil fuel recovery with research to study sequestration on a large scale.

What’s needed now, says Jim Katzer, a visiting scholar at MIT’s Laboratory for Energy and the Environment, are more large-scale demonstrations of CCS in multiple geologies, integrated with policies that address site selection, licensing, liability, and other issues. Katzer says there are a number of investigations that are investigating storage in the 5,000- to 20,000-ton-capacity range, and they’re generating some useful information. “But,” he says, “none of them are getting us to the answer we really need: how are we going to manage storage in the millions of tons over long periods of time?”

## Paying for Storage

The task of managing carbon storage is nothing if not daunting: in the United States alone, coal plants produce more than 1.5 billion tons of CO_2_ every year. Sequestering that amount of gas will require not only a vast new infrastructure of pipelines and storage sites but also that the country’s coal plants adopt costly technologies for carbon capture. Most existing U.S. plants—indeed, most of the world’s 5,000 coal-fired power plants, including the ones now being built in China—burn pulverized coal (PC) using technologies essentially unchanged since the Industrial Revolution. CO_2_ can be extracted from PC plants only after the fuel has been burned, which is inefficient because the combustion emissions are highly diluted with air.

A more efficient approach is to capture highly concentrated streams of CO_2_ from coal before it’s burned. Precombustion capture is usually applied at integrated gasification combined cycle (IGCC) coal plants, which are extremely rare, numbering just five worldwide, according to Sarkus. IGCC plants cost roughly 20% more to operate because the gasification process requires additional power, which explains why there are so few of them.

Although they don’t rule out the possibility, none of the industry sources interviewed for this article welcome the prospect of retrofitting traditional PC plants for carbon capture. That would require major plant modifications and could potentially double the cost of electricity to consumers, they say. But by ignoring existing facilities, industry will set back CCS expansion by decades—most PC plants in use today have been designed for lifetimes of 30 to 40 years.

Whatever path it takes, the transition to CCS will require enormous sums of money. When used for enhanced recovery, CO_2_ is a commodity that pays for its own burial. But only a small fraction of the CO_2_ generated by coal plants and other industrial processes is used for that purpose. Creating a broad CCS infrastructure will ultimately require a charge on carbon emissions that, according to calculations described in *The Future of Coal*, should total at least $30 per ton—$25 per ton for CO_2_ capture and pressurization and $5 per ton for transportation and storage—with this figure rising annually in accordance with inflation.

Sally Benson, a professor of energy resources engineering at Stanford University, points to different ways to pay that charge. One is a tax on CO_2_ emissions, an option she concedes has little political support. Funds could also be raised with a “cap-and-trade” system, which sets area-wide limits on CO_2_ emissions that industries can meet by trading carbon credits on the open market. A cap-and-trade system for CO_2_ has already been established by the European Union, which regulates the greenhouse gas to meet obligations under the Kyoto Protocol. Jeff Chapman, chief executive officer of the Carbon Capture and Storage Association, a trade group based in London, suggests the European cap-and-trade system could ultimately raise €62 billion.

In the United States, a national cap-and-trade system likely won’t appear until the federal government regulates CO_2_ as a pollutant, says Luke Popovich, vice president of external communications with the National Mining Association, a coal industry trade group in Washington, DC. In the meantime, individual states—for instance, California, which sets its own air quality standards per a waiver under the Clean Air Act—are planning for their own cap-and-trade systems. California regulates CO_2_ under a state law called AB32, which directs industries to reduce all greenhouse gas emissions by 25% over the next 13 years. CCS may ultimately emerge on a state-by-state basis in this country, where charges on carbon emissions allow it, Benson suggests.

None of [the CCS studies under way] are getting us to the answer we really need: how are we going to manage storage in the millions of tons over long periods of time?

–Jim Katzer, Massachusetts Institute of Technology

## Technical Questions Remain

Until the early 1990s, most researchers involved in CCS worked in isolation. But in March 1992, more than 250 gathered for the first International Conference on Carbon Dioxide Removal in Amsterdam. Howard Herzog, a principal research engineer at the MIT Laboratory for Energy and the Environment and a leading expert on CCS, says attendees arrived as individuals but left as a research community that now includes funding agencies, industries, and nongovernmental organizations throughout the world. Unfortunately, that community doesn’t have nearly the resources it needs to study CCS on a realistic scale, Katzer says. Indeed, *The Future of Coal* states emphatically that “government and private-sector programs to implement on a timely basis the large-scale integrated demonstrations needed to confirm the suitability of carbon sequestration are completely inadequate.”

Absent sufficient evidence, most experts simply assume that vast amounts of sequestered CO_2_ will stay in place without leaking to the atmosphere. They base that assumption on available monitoring data from the big three industrial projects—none of which have shown any evidence of CO_2_ leakage from their underground storage sites, according to *The Future of Coal*—and also on expectations that buried CO_2_ will behave in essentially the same way as underground fossil fuel deposits. “We’re optimistic it will work,” says Jeffrey Logan, a senior associate in the Climate, Energy, and Transport Program at the World Resources Institute. “The general theory is that if oil and gas resources can remain trapped for millions of years, then why not CO_2_?”

Franklin Orr, director of the Global Climate and Energy Project at Stanford University, says monitoring data show that CO_2_ injected underground for enhanced oil and gas recovery remains trapped there by the same geological structures that trapped the fuels for millions of years; specifically overlying shale deposits through which neither fossil fuels nor CO_2_ can pass. Decades of research by the oil and gas industries, in addition to basic research in geology, have revealed the features needed for CO_2_ sequestration, he says: “You’re looking for deep zones with highly porous rocks—for instance, sandstone—capped by shale seals with low permeability. Sleipner and Weyburn are both good examples; both have thick shale caps that keep the CO_2_ from getting out.”

But Orr concedes that questions remain about how large amounts of CO_2_ might behave underground. A key risk to avoid, he says, is leakage through underlying faults or abandoned wells that provide conduits to the atmosphere. Yousif Kharaka, a research hydrologist with the USGS in Menlo Park, California, says an unknown but possibly large number of orphaned or abandoned wells in the United States could pose a risk of leakage to the atmosphere. And that, he warns, would negate the climate benefits of sequestration.

The likelihood that CO_2_ levels could accumulate and cause health or ecological injuries is minimal, Kharaka says, echoing the conclusions reached in *The Future of Coal*. He says CO_2_ in air only becomes harmful to humans at concentrations of 3% or above, which is far higher than might be expected from slow leaks out of the ground. Nonetheless, the possibility that CO_2_ leaking from underground storage sites might accumulate to harmful levels in basements or other enclosed spaces can’t be discounted entirely, cautions Susan Hovorka, a senior research scientist at the Bureau of Economic Geology, a state-sponsored research unit at the University of Texas at Austin. “It’s important that we manage this substance correctly,” she says. “If you determine that there’s a risk to confined places, then you have to provide adequate ventilation. But we have a high level of confidence that CO_2_ will be retained at depth.”

The greater concern says Kharaka, is that migrating CO_2_ might mix with brine, forming carbonic acid that could leach metals such as iron, zinc, or lead from the underlying rock. In some cases, acidified brine alone could migrate and mix with fresh groundwater, posing health risks through drinking or irrigation water, he says. Results from an investigation conducted near Houston, Texas, led by Hovorka as principal investigator along with Kharaka and other scientists from 21 organizations, indicate that CO_2_ injected into saline aquifers produced sharp drops in brine pH, from 6.5 to around 3.5. These results were published in the September 2007 report *Water–Rock Interaction: Proceedings of the 12th International Symposium on Water–Rock Interaction, Kunming, China, 31 July–5 August, 2007*. Chemical analyses showed the brine contained high concentrations of iron and manganese, which suggests toxic metal contamination can’t be ruled out, Hovorka says. “I’d describe this as a nonzero concern,” she adds. “It’s not something we should write off, but it’s not a showstopper.” [For more on public perception of CCS hazards, see “Of Two Minds: Groups Square Off on Carbon Mitigation,” p. A546 this issue.]

Coal companies are hoping to build new plants before cap-and-trade regulations go into effect—and they will, soon—with the idea that the plants and their greenhouse gas emissions will be grandfathered in until sequestration is technically and financially feasible. This is an enormously risky investment decision on their part, and morally irresponsible.

–Leslie Harroun, Oak Foundation

Experts in this area consistently point to the need for more detailed investigations of CO_2_ movements at depth and their geochemical consequences. Hovorka’s investigation was among the first of this kind, but its scale—just 1,600 tons—paled in comparison to realistic demands for CO_2_ mitigation to combat climate change.

Constrained by inadequate funding, the DOE has put much of its CCS investment into a project dubbed “FutureGen.” This initiative seeks to build a prototype coal-fired power plant that will integrate all three features of a CCS system, namely, carbon capture (achieved with IGCC technology), CO_2_ transportation, and sequestration. Supported by the DOE and an alliance of industry partners, the four-year, $1.5 billion project was announced formally by President Bush in his 2002 State of the Union Address. Once operational, the plant will supply 275 megawatts of power (compared with the 600–1,300 megawatts supplied by typical U.S. coal plants), enough for 275,000 households. Sarkus, who is also the FutureGen director, says four potential sites for the plant and its CO_2_ reservoirs—including two in Illinois and two in Texas—are under consideration. Final site selection, he says, will depend on community support, adequate transportation lines, and proximity to underground storage reservoirs.

The Bush administration’s stance is that FutureGen will promote CCS advancements throughout the coal and utility industries. But many stakeholders don’t think it goes far enough toward meeting existing needs; the project is “too much ‘future’ and not enough ‘generation,’” quips Hawkins. “What we need is legislation that specifies future power plants must be outfitted with CCS, period.” To that, Katzer adds, “FutureGen was announced in 2002, and they still haven’t settled on site selection, nor have they resolved key design issues. Operations were set to begin in 2012, and now that’s slipping back even further. Assuming you start in 2012 and operate for four years, you’re looking at 2016 before you complete a single demonstration project. That stretches things out too far, and speaks to the need for several demonstration projects funded now by the U.S. government so we can deal with CO_2_ emissions in a timely fashion.”

## The Developing Country Factor

With U.S. research efforts stuck in low gear, concerns over a comparable lack of progress in the developing world are growing. China already obtains more than 80% of its domestic electricity from coal. And with a relentless push for economic growth, lowering CO_2_ emissions from its coal plants is a low priority. It’s likely that none of China’s coal-fired plants are outfitted for carbon capture, says Richard Lester, a professor of nuclear science and engineering at MIT. “Given the scale and expansion of China’s electric power sector, the eventual introduction of CCS there is going to be absolutely critical to global efforts to abate or reduce the atmospheric carbon burden,” he says.

Meanwhile, India lags just a decade or less behind China in terms of its own economic growth, which is increasingly fueled by coal use, Katzer says. The key difference between the two countries, he says, has to do with planning for environmental and energy development. In China, Katzer explains, growth and environment strategies seem to be dictated at regional levels without any central coordination, which is ironic considering the country’s socialist political structure. India, on the other hand, seems to have what Katzer calls a “master plan” for growth. “But they have no clue how to move forward in terms of CO_2_ reductions,” Katzer says. “What officials in India say to me is, ‘We’ll manage CO_2_ if it doesn’t cost too much.’ That’s the downside in all of this.”

In the end, CCS seems to be stuck in a catch-22: In the view of the developing world, the United States and other wealthier nations should take the lead with respect to emissions reduction technology. Governments in wealthier nations, meanwhile—particularly the United States—look to industries in the free market for solutions to the problem. But U.S. industries say they can’t afford large-scale research; in industry’s opinion, the government should pay for additional studies that lay the groundwork for industry research and the technology’s future implementation. The government, however, doesn’t fund the DOE and other agencies at nearly the amounts required to achieve this. And at the same time, the two mechanisms that could possibly generate sufficient revenues for CCS—carbon taxes and cap-and-trade systems for CO_2_ emissions—are trapped by perpetual political gridlock.

Leslie Harroun, a senior program officer at the Oak Foundation, a Geneva-based organization that funds social and environmental research, warns that industry might leverage the promise of CCS as a public relations strategy today while doing little to ensure its broad-based deployment tomorrow. “The coal industry’s many proposals to build ‘clean’ coal plants that are ‘capture ready’ across the U.S. is a smokescreen,” she asserts. “Coal companies are hoping to build new plants before cap-and-trade regulations go into effect—and they will, soon—with the idea that the plants and their greenhouse gas emissions will be grandfathered in until sequestration is technically and financially feasible. This is an enormously risky investment decision on their part, and morally irresponsible, but maybe they think there is power in numbers.”

In a sense, the inertia surrounding CCS might reflect a collective wilt in the face of a seemingly overwhelming technical and social challenge. To make a difference for climate change, a CCS infrastructure will have to capture and store many billions of tons of CO_2_ throughout the world for hundreds of years. Those buried deposits will have to be monitored by unknown entities far into the future. Many questions remain about who will “own” these deposits and thereby assume responsibility for their long-term storage. Meanwhile, industry and the government are at an impasse, with neither taking a leading role toward making large-scale CCS a reality. How this state of affairs ultimately plays out for health of the planet remains to be seen.

## Whatever Happened to Deep Ocean Storage?

One CCS option that appears to have fallen by the wayside is deep ocean storage. Scientists have long speculated that enormous volumes of CO_2_ could be stored in the ocean at depths of 3 km or more. High pressure would compress the CO_2_, making it denser than seawater and thus enabling it to sink. So-called CO_2_ lakes would hover over the sea floor, suggests Ken Caldeira, a Stanford University professor of global ecology.

“A coal-fired power plant produces a little under one kilogram of CO_2_ for each kilowatt-hour of electricity produced,” says Caldeira. “An individual one-gigawatt coal-fired power plant, . . . if completely captured and the CO_2_ stored on the sea floor, would make a lake ten meters deep and nearly one kilometer square—and it [would grow] by that much each year.”

But Caldeira and others acknowledge that deep ocean storage doesn’t offer a permanent solution. Unless the gas is somehow physically confined, over time—perhaps 500 to 1,000 years—up to half the CO_2_ would diffuse through the ocean and be released back into the atmosphere. Moreover, most life within CO_2_ lakes would be extinguished. However, Caldeira believes this consequence would be balanced by the benefits of keeping the greenhouse gas out of the atmosphere, where under global warming scenarios it acidifies and endangers sea life at the surface.

No one knows precisely what would happen during deep ocean storage because it’s never been tested. A planned experiment off the coast of Hawaii in the late 1990s, with participation of U.S., Norwegian, Canadian, and Australian researchers, was canceled because of opposition of local environmental activists. According to Caldeira, who previously co-directed the DOE’s now-defunct Center for Research on Ocean Carbon Sequestration, government program managers who backed the Hawaiian study were laterally transferred, sending a signal that advocating for this type of research was politically dangerous for career bureaucrats. “Today, there’s zero money going into it,” Caldeira says. “Right now, ocean sequestration is dead in the water.”

## Figures and Tables

**Figure:**
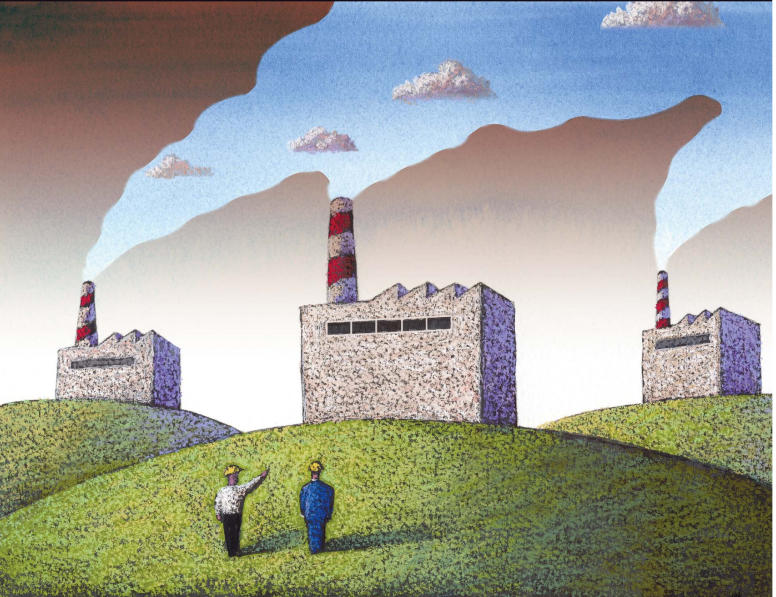


**Figure:**
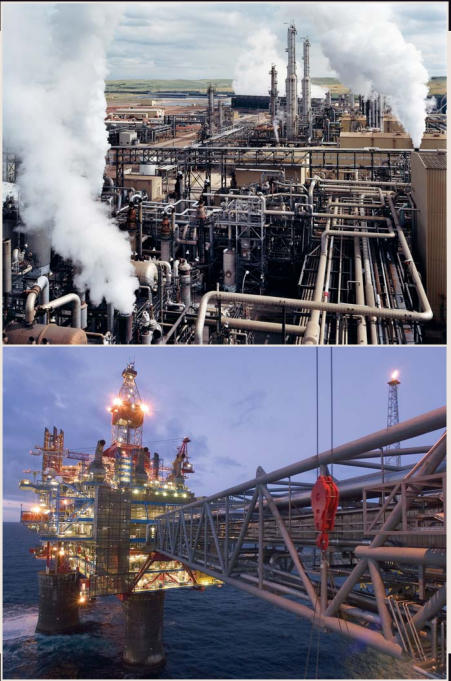
There are currently three industrial-scale CCS operations under way around the world: the Great Plains Synfuel Plant in Beulah, North Dakota (top), which ships its CO2 200 miles away for use in enhanced recovery; Norway's Sleipner West gas platform (above), which buries its emissions under the sea floor; and the In Salah project in Algeria (not pictured), which sequesters CO2 underground.

**Figure:**